# The Role of Stress in Absenteeism: Cortisol Responsiveness among Patients on Long-Term Sick Leave

**DOI:** 10.1371/journal.pone.0096048

**Published:** 2014-05-02

**Authors:** Henrik B. Jacobsen, Johan Håkon Bjørngaard, Karen W. Hara, Petter C. Borchgrevink, Astrid Woodhouse, Nils Inge Landrø, Anette Harris, Tore C. Stiles

**Affiliations:** 1 Department of Circulation and Medical Imaging, Norwegian University of Science and Technology, Trondheim, Norway; 2 Hysnes Rehabilitation Center, St. Olav's University Hospital, Trondheim, Norway; 3 National Centre for Complex Disorders, St. Olav's University Hospital, Trondheim, Norway; 4 Department of Public Health and General Practice, Norwegian University of Science and Technology, Trondheim, Norway; 5 Forensic Department and Research Centre Brøset, St. Olav's University Hospital, Trondheim, Norway; 6 Clinical Neuroscience Research Group, Department of Psychology, University of Oslo, Oslo, Norway; 7 Department of Health Promotion and Development, Faculty of Psychology, University of Bergen, Bergen, Norway; 8 Department of Psychology, Norwegian University of Science and Technology, Trondheim, Norway; John Hopkins University School of Medicine, United States of America

## Abstract

**Objective:**

This study aimed to (1) See whether increased or decreased variation relate to subjective reports of common somatic and psychological symptoms for a population on long-term sick leave; and (2) See if this pattern in variation is correlated with autonomic activation and psychological appraisal.

**Methods:**

Our participants (n = 87) were referred to a 3.5-week return-to-work rehabilitation program, and had been on paid sick leave >8 weeks due to musculoskeletal pain, fatigue and/or common mental disorders. An extensive survey was completed, addressing socio-demographics, somatic and psychological complaints. In addition, a physician and a psychologist examined the participants, determining baseline heart rate, medication use and SCID-I diagnoses. During the 3.5-week program, the participants completed the Trier Social Stress Test for Groups. Participants wore heart rate monitors and filled out Visual Analogue Scales during the TSST-G.

**Results:**

Our participants presented a low cortisol variation, with mixed model analyses showing a maximal increase in free saliva cortisol of 26% (95% CI, 0.21–0.32). Simultaneously, the increase in heart rate and Visual Analogue Scales was substantial, indicating autonomic and psychological activation consistent with intense stress from the Trier Social Stress Test for Groups.

**Conclusions:**

The current findings are the first description of a blunted cortisol response in a heterogeneous group of patients on long-term sick leave. The results suggest lack of cortisol reactivity as a possible biological link involved in the pathway between stress, sustained activation and long-term sick leave.

## Introduction

The majority of long-term sick leave is explained with musculoskeletal pain and mental disorders where stress management and job strain might play an important role [1]. The hypothalamus-pituitary-adrenal (HPA) axis and its end product, cortisol, has been linked to chronic pain, chronic fatigue, anxiety and depression [2]–[5]. These four symptoms often justify long-term sick absence and decrease the individuals' quality of life [1], [6].

Stress however, either in the form of job strain or other negative psychosocial experiences, is a widely debated concept with numerous definitions [7]. A recent systematic review argues that a strict definition is needed, and that only a physiological definition of stress characterized by neuroendocrine reactions can provide this [7]. Incorporating appraisal and experience, Koolhaas et al., [7] argue that pathological stress occurs only when the individuals' demands exceed their normal regulatory capacity over time. This is marked by the lack of an anticipatory neuroendocrine response and/or a reduced neuroendocrine recovery [7]. This definition is supported by accumulated data from human studies demonstrating how persistent physiological stress affect the feedback mechanisms of the HPA-axis [8].

A recurring problem with cortisol studies is that the findings are somewhat contradictory, often showing both hypo and hyper activation of the HPA-axis [2], [8]. Along with the aforementioned review, several studies have suggested that contradictory results are related to sustained activation of the HPA-axis over time, yielding different phases of dysregulation [7], [9]. This would entail that dysregulation can create both a hypo- or hyper expression of cortisol in the same phenotype, the common factor being a lack of variability and/or reactivity in the hormonal expression [3], [8], [10]. Low variability in cortisol release combined with reduced habituation has been suggested as a direct physiological expression of vital exhaustion, a mental state in which the catabolic capacity for stress adaptation is disrupted [10]–[12]. This hypothesis has received support from several studies using a standardized psychosocial stress procedure known as the Trier social stress test [3], [13]. In healthy participants, the Trier test has been administrated more than 4000 times and is consistently shown to predict stress in the form of saliva cortisol increase [13], [14]. A meta-analysis of 48 Trier test studies on healthy participants showed a large mean cortisol effect size of 0.92 (95% confidence interval (CI) 0.70, 1.14) and this effect proved highly significant (*p*<0.001). Almost the same effect size was observed for recovery after the stressor had been completed, describing almost an inverted U relationship (*d* = 0.85, 95% CI 0.63, 1.07, *p*<.01) [15].

With cortisol being a metabolic hormone with the critical function of mobilizing energy and reducing inflammation, *one* suggested pathway or mechanism could be that sustained physiological stress activation may be causally related to fatigue, musculoskeletal pain, depression, and anxiety through this lack of variability [12].

Describing evidence for such a pathway more specifically, several studies show a link between life stress, hyper vigilance and development of fibromyalgia [16]. This is supported by results showing that altered HPA-activity is strongly associated with both regional and widespread pain [4]. Depressive symptoms are linked to life stress to such an extent that a causal relationship has been suggested [17]. A potential mechanism explaining this link has been demonstrated through reduced glucocorticoid receptor sensitivity modulated by a polymorphic expression of the FKBP5 gene [18]. This is also demonstrated in longitudinal studies of cortisol secretion, where low variation in diurnal cortisol release has been linked with depression [19], and depressed patients demonstrate both hyper- and hypo-activation of the HPA-axis [5]. Similar findings are reported in a range of anxiety disorders [15], [18], where a low variability of cortisol expression in response to the Trier social stress test in patients with panic disorder, is perhaps the most striking finding [3]. In chronic fatigue syndrome, stress has been shown to exacerbate the symptoms of fatigue [20], and these patients also demonstrate blunted responses to a standardized psychosocial stressor [21].

The contradiction of having both hypo- and hypercorticolism findings is often highlighted and possibly caused by numerous confounding variables, like differences in methods and/or population characteristics [8], [22]. It has been suggested that for disorders like pain and fatigue, one of the most important confounding factors is psychiatric comorbidity [23]. The current study specifically addresses psychiatric disorders and confounding variables by providing an extensive somatic, psychological and social screening.

Combining basic research in endocrinology, psychology and clinical studies, we wanted to utilize the knowledge about such pathways to better understand the major causes of disability and investigate potential common factors. The aims of this study are: 1) when controlling for socio-demographics, to use a standardized stressor to see whether increased or decreased cortisol variation is related to SCID-diagnoses, current medication and/or subjective reports of somatic and psychological symptoms in a population on long-term sick leave; and 2) to see if this pattern in variation is related to autonomic activation and/or psychological appraisal.

We hypothesize that our participants will display a reduced variation in cortisol secretion when responding to a standardized psychosocial stress test, regardless of their self-reported symptom load and autonomic activation.

## Materials and Methods

### Ethics statement

All participants signed an informed consent outlining the study before inclusion.

The study was approved by the Regional Committee for medical and health research ethics, Central Norway and conducted in accordance with the Declaration of Helsinki.

### Study participants

This was a repeated measures study with participants being consecutively recruited from a clinical setting throughout 2012. General practitioners (GP) referred patients between 18–59 years of age to a 3.5-week in-patient intervention at Hysnes Rehabilitation Center, Norway. The patients had been on sick leave longer than 8 weeks due to musculoskeletal pain, fatigue and/or common mental disorders. Prior to the intervention, the participants filled out an extensive web-based survey provided by CheckWare. This survey included measures of socio-demographics, pain, fatigue, mental distress and sleep problems. Participants also met with a physician and a psychologist for psychological and physical examinations. Upon admission, the participants were asked to participate in a study while receiving the intervention. A total of 194 patients were included in a main study investigating the effects of rehabilitation. From this population, 87 patients were selected via list randomization to participate in a stress study. The stress study involved participating in standardized stress tests pre- and post-intervention.

Participants were excluded only if they had acute psychosis, ongoing manic episode or suicidal ideation, were not able to communicate in Norwegian, or if they were pregnant. Also, this being return-to-work rehabilitation, participants were excluded if did not define return to work as a personal goal. Of the 87 participants in the stress study, 3 participants were coded as missing because of missing values on the key variable cortisol, leaving the final *n* at 84 participants.

### Standardized stress procedure

To create acute psychosocial stress we used the Trier Social Stress Test for Groups [24]. This protocol has repeatedly demonstrated the ability to elicit a significant stress response in healthy participants [24], [25]. This test deviates from the original Trier Social Stress Test on actual exposure time (2 versus 5 or 10 minutes), but has been validated against the Trier Social Stress Test in several studies [24], [25].

**Figure 1:**
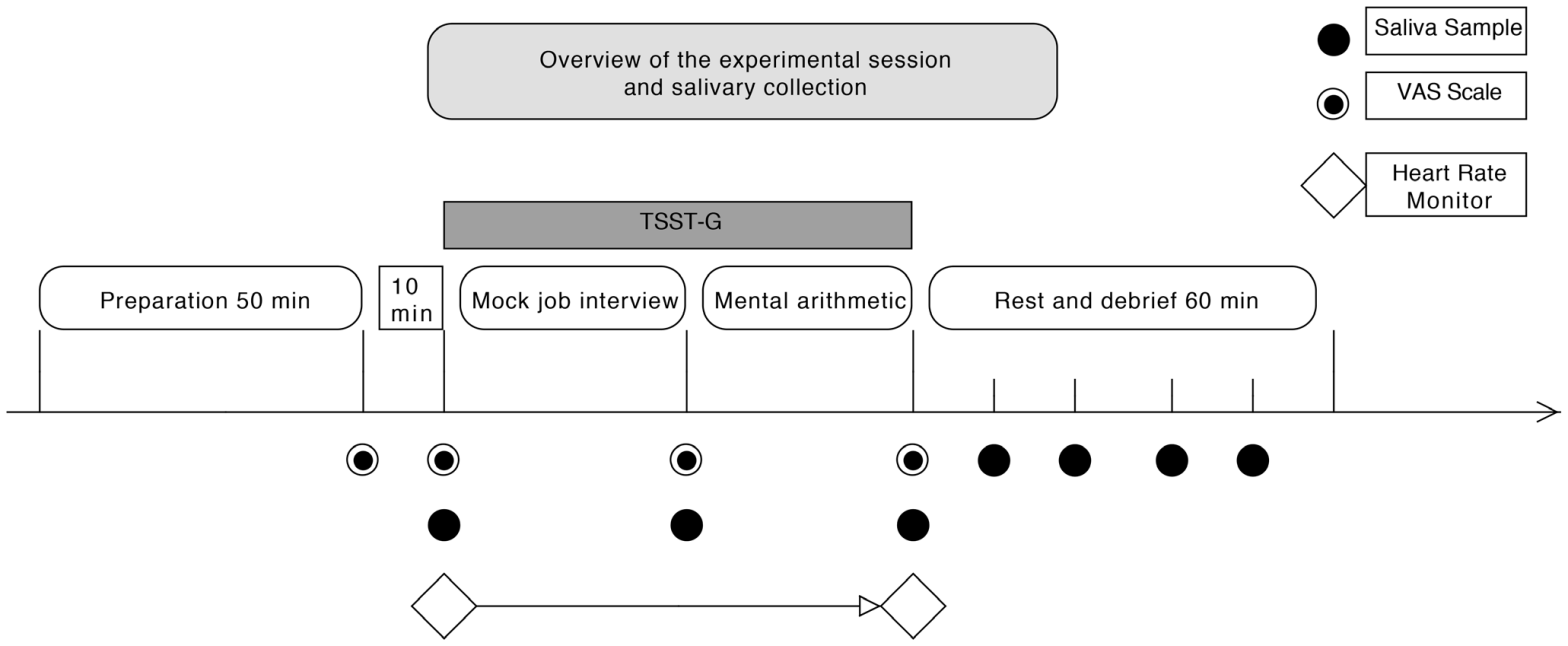
Overview of the exposure session showing collection of saliva samples, VAS scales and heart rate monitoring.

The Trier Social Stress Test for Group (TSST-G) consists of a mock job interview and mental arithmetic performed in front of a group of peers and an experimenter panel. Each session lasts approximately 2,5 h, including a 60-minute *preparation phase*, a 20-minute *exposure phase* and a 60-minute *recovery phase*. Each test includes 7 measure-points of saliva cortisol collected by the research personnel pertaining to the timeline illustrated in figure 1. All TSST-G exposures were conducted at one of two time slots, either 1630 h to 1900 h or 1300 h to 1530 h. These time slots have been statisticly validated through rigouros studies [26].

In the *preparation phase*, the participants were told to relax and avoid any strenuous activity. Ten minutes before the preparation phase was over the participants were told to prepare for a job interview, and filled out three *Visual Analog Scales (VAS)*. Afterwards the participants entered the *exposure phase*, where each participant was given a number so that they could be identified during the experiment. Mobile walls were used to separate the participants, and they received no feedback other than the standardized instructions from a designated spokesperson in the test panel. The *exposure phase* was initiated with a two-minute random exposure through the public speaking task/job interview and after all participants had performed the interview, and taken a second saliva sample, a mental arithmetic task was introduced. Each participant was then randomly selected to do arithmetics for 1.5 minutes as part of the *exposure phase*. After completion of the arithmetics the test moved over in the *debriefing phase*, where the participants were given the opportunity to share thoughts and reflections about their experience. A saliva sample was taken every 15 minutes of the debriefing period (in total 4 salivettes) to monitor recovery. An overview of the entire experimental session with sampling is illustrated in figure 1, and further details about the experimental protocol are available in the appendix S1.

### Endocrine stress responses

The sampling of saliva cortisol followed earlier protocols studying cortisol responses to acute stress [24]. The saliva samples were collected with purpose-designed polyester salivettes (Sarstedt Inc., Rommelsdorf, Germany), which has been used in several previous studies [24], [27]. After sampling, the salivettes were immediately frozen at −20 °C.

For analyses, at the Department of Medical Biochemistry at St. Olav's Hospital, Trondheim, the samples were thawed, centrifuged and analyzed on Modular E170 from Roche using an electrochemiluminescens immunoassay (ECLIA) method. Modular E 170 is a fully automatic analysis module for immunochemical assays. The assay used for determination of cortisol in saliva had an interassay variability of 7.9% at 12 nmol/L.

### Autonomic stress responses

Continuous recording of heart rates was used both as a measure of task engagement and sympathetic arousal. This study used a wireless chest heart rate transmitter and a wristwatch recorder (Polar RS800TM, Polar Electro, Finland). A trained physician assessed a baseline recording of heart rate during the examination at Hysnes outpatient clinic. The subjects were standing in an upright position during the baseline recording and all through the exposure phase of the TSST-G.

### Self-reported somatic and psychological symptoms

*Chronic fatigue* was measured with the Chalder Fatigue Questionnaire [28] that consists of eleven questions assessing physical and mental fatigue. The scale was later revised to include two items pertaining to extent and duration [29]. Each item has four response categories scored bimodally 0-0-1-1. The 11 first items are summed and yield a scale of 0–11. This thirteen-item scale has been validated for a Norwegian population, and has a cut-off on symptom intensity ≥5, lasting for 6 months or more [29].

The Hospital Anxiety and Depression Scale (HADS) [30] assesses symptoms of *anxiety and depression.* The fourteen-item scale with each item ranging from 0–3 yields separate scores for anxiety and depression, which are summed. A score ≥8 on either subscale was used to indicate caseness. This cut-off is validated for a Norwegian population [31].

*Chronic pain* was measured with an item from Short Form-8 [32] asking “How much bodily pain have you had the last week? (None, very mild, mild, moderate, severe, very severe)”. This scale has been validated as a self-report measure of chronic pain in Norwegian population studies, using the cut-off ≥moderate [33].

The Insomnia Severity Index [34] (ISI) is a seven-item questionnaire assessing *sleep problems*. A 5-point (0-4) scale, rated difficulties falling asleep, night-time awakenings, early morning awakenings, impairment of daytime functioning due to sleep problems, notice ability of impairments, distress or worry caused by sleep difficulties, and dissatisfaction with sleep. The items were summed; giving a scale of 0–28, where ≥15 was used as a cut-off indicating caseness. This cut-off has been validated in previous studies [35].

The participant's *age, gender, height, weight, relationship status, and education* were reported through a standardized set of questions described elsewhere [36].

The participants filled out *Visual Analog Scales (VAS)* 10 minutes before, and 3 times during, the *exposure phase* of TSST-G, as done in previous studies [24]. These scales held one question each with a range 0–100, 0 being “not at all”, and a 100 being “the most intense experience tolerable”. The statements pertained to avoidance “To what extent do you want to leave this setting?” Anxiety: “To what extent do you feel uncomfortable in this setting?” And tension: “To what extent do you feel tension in your body right now?” VAS scales are considered a valid and feasible measure of clinical phenomena in an experimental setting [37].

### Psychological and medical examination

Using the Structured Clinical Interview for DSM-IV [38] (SCID-I), a clinical psychologist screened participants for the 13 most common psychiatric disorders. Using criteria from DSM-IV, the diagnoses ranged from depression to panic disorder. In brief, the following diagnoses were present: Major Depression (6 cases), Recurrent Depression (3 cases), Dysthymia (2 cases), Panic Disorder (2 cases), Anxiety (3 cases) and Post Traumatic Stress Disorder (1 case).

A physician reviewed the participants' medical records and assessed current medication and smoking, adding this to the research database. *Current smoker* was defined as having one or more cigarettes the last 7 days, *current medication* was defined as a valid prescription at the time of examination. Antidepressants and synthetic hormones were included as potential confounders based on a review investigating medications affecting saliva cortisol secretion [39].

### Statistical analyses

Free saliva cortisol values in the TSST-G were modeled by repeated measures using the natural log transformation of cortisol and fitting random effects models with random slopes [40]. The results were transformed back to yield values in nmol/l. The participant's sex, age and BMI were fixed on average levels to represent mean values for this population. Autonomic activation was analyzed through a paired t-test comparing average heart rate during an examination at the outpatient clinic with heart rate during the TSST-G exposure. Scores on Visual Analogue Scales were modeled for repeated measures and we used random effects models fitted with random slopes as with the saliva cortisol measures.

All results are reported with a coefficient and/or average values with 95% confidence intervals (CI). All analyses were carried out in Stata, version 12.0 (Stata Corporation, Inc, College Station, TX).

## Results

### Participant characteristics

The participants (N = 84) were 71.4% women with a mean age of 41.5 years (SD, 10.0 years). The majority was married or living with a partner (71%) and had some college or university education (54.9%). Seventy-nine percent of our participants reported fatigue lasting more than 6 months, 75% reported chronic pain, 54% reported depression, 61% reported anxiety and 46% reported more than 3 symptoms above the clinical cut-off (Table 1).

**Table 1:** Characteristics of participants (n = 84) included in the Trier Social Stress Test for Groups.

Demographics	Male	Female
Gender (n)	28.6% (24)	71.4% (60)
Age (SD)	41.0 (11.8)	41.1 (9.2)
Body Mass Index (SD)	27.3 (4.6)	26.7 (6.8)
Married/Living with Partner (n)	68.2% (15)	71.7% (43)
Single/Divorced/Widowed (n)	31.8% (7)	28.3% (17)
Education (n):		
Less than High School	18.2% (4)	11.9% (7)
High School	22.7% (5)	35.6% (21)
Some College/University	54.5% (12)	35.6% (21)
Any College/University Degree	4.5% (1)	16.9% (10)
Medication*(n):		
Synthetic Thyroid Hormone	0	20% (12)
Antidepressants/Beta Blockers	13.6% (3)	6.6% (4)
Current Smoker (n)	40.9% (9)	10.3% (6)
SCID-I Diagnoses (n)	21% (5)	20% (12)
**Self-reported Caseness**	**Male**	**Female**
Depression (n)	54.5% (12)	53.3% (32)
Anxiety (n)	54.5% (12)	67.7% (40)
Chronic Fatigue (n)	77.3% (17)	76.6% (46)
Chronic Pain (n)	72.7% (16)	78.3% (47)
Sleep Problems (n)	31.8% (7)	45.8% (27)
More than 3 symptoms above cut-off	45,8% (11)	45,8% (27)

Categorical characteristics are reported as percent (frequency) and ordinal characteristics as mean (standard deviation).

*Of these participants, some received more than one of the three medications listed. Numbers may not add up to 84 because of missing data on some characteristics

**Figure 2:**
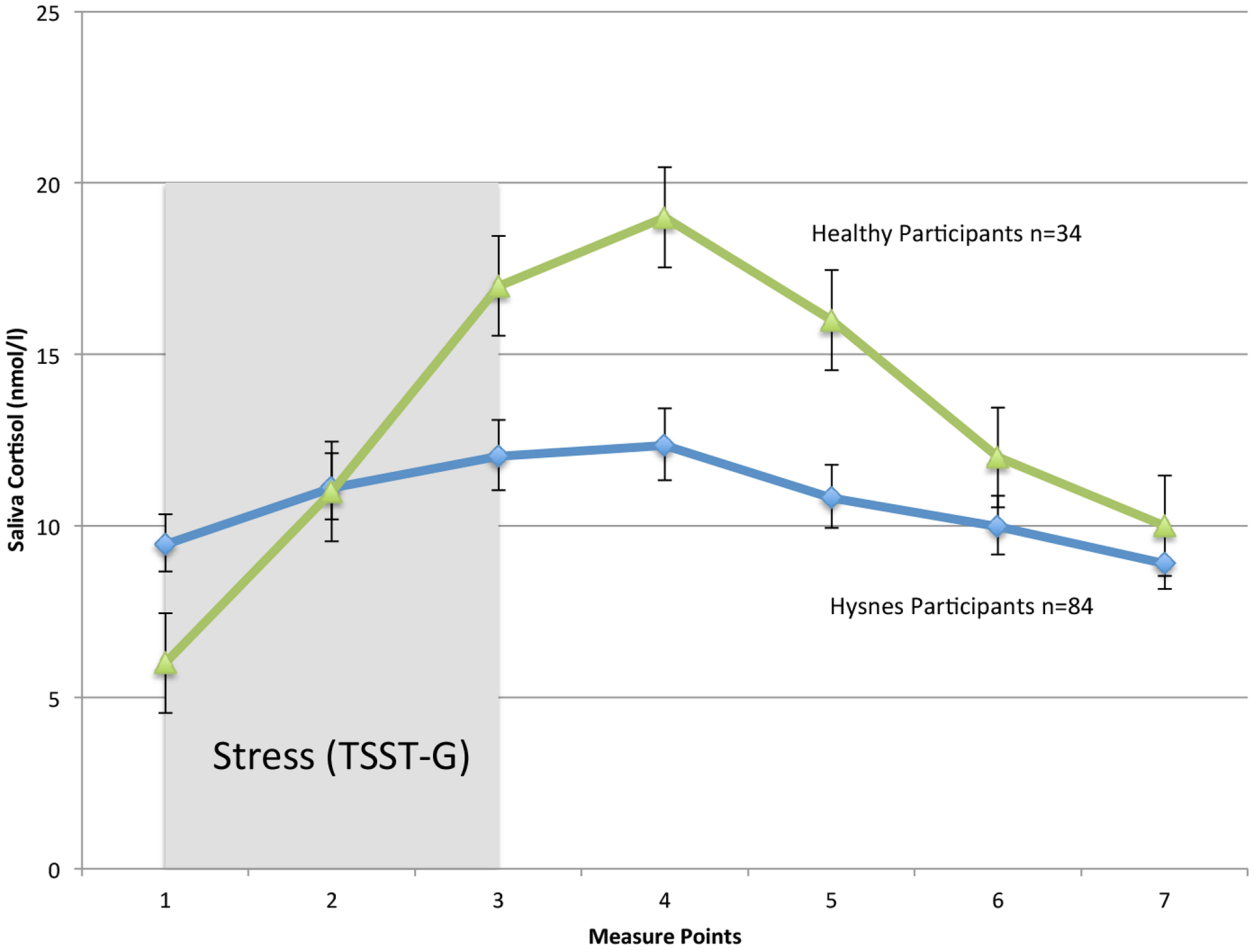
Cortisol responses to a standardized psychosocial stress test (TSST-G) in participants on long-term sick leave attending Hysnes Rehabilitation Center (n = 84) and healthy subjects (n = 34) adapted from von Dawans et al [24] (licensed reuse by Elsevier, license number: 3175410277640) exposed to identical experimental procedures (TSST-G). The gray bar represents the TSST-G exposure phase, and error bars represent 95% confidence intervals.

The participants in this study presented a low cortisol variation in response to the TSST-G. Our participants showed a 26% (95% CI, 0.21–0.32) higher cortisol level at the 4^th^ time point, when compared with the first (Table 2). Investigating recovery, our participants did not show any substantial recovery during the exposure phase or the rest and debrief phase (−.06%, 95% CI, −0.12–.004) (Figure 2).

**Table 2:** Averaged variation of saliva cortisol compared measure point 1 with sex, BMI and age fixed at averaged levels for this population.

Measure Point	Coefficient	95%, CI	
1	Comparison		
2	0.16	0.11	0.21
3	0.24	0.19	0.29
4	0.27	0.21	0.32
5	0.13	0.08	0.19
6	0.05	−0.008	0.11
7	−0.06	−0.13	0.004

Coefficients represent % rise or fall compared with the initial value at the start of exposure to TSST-G.

**Figure 3:**
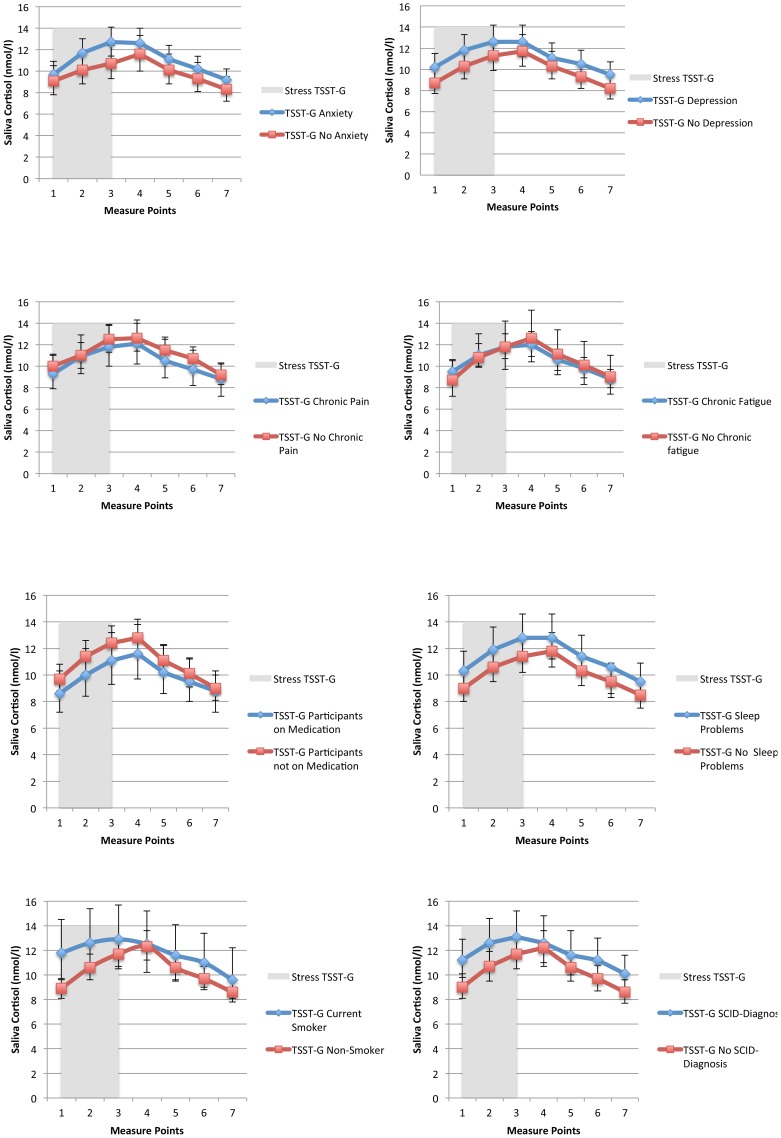
Fitted random effects models of subgroup analyses on participant characteristics. Characteristics were dichotomized as “Yes” or “No” based on established cut-offs (fatigue, pain, anxiety, depression and/or sleep problems) or occurrence/non-occurrence (smoking, SCID-diagnosis and/or medication). The gray bar represents the TSST-G exposure phase, and error bars represent 95% confidence intervals.

In figure 3, TSST-G curves from participants were dichotomized as “Yes” or “No” based on established cut-offs (fatigue, pain, anxiety, depression and/or sleep problems) or occurrence/non-occurrence (smoking, SCID-diagnosis and/or medication). There was weak evidence for any statistical interaction between the exposure effect on cortisol response and self-reported caseness of chronic fatigue, depression, anxiety, chronic pain, sleep problems, antidepressant/synthetic hormone medication, one or more SCID Axis-I diagnoses, age, sex or BMI (p>0.05). There was evidence of statistical interaction between the exposure effect on cortisol response and smoking (p = 0.003). There was a tendency towards a higher initial cortisol level and a weaker exposure effect in smokers (Figure 3).

### Heart rate stress responses

Our participants demonstrated an averaged increase of 41.2 beats/min (95% CI, 36.8–45.5) from the baseline measure to the TSST-G exposure phase. This reflects a significant autonomic activation when comparing a control measure done by a physician with the 20 minutes of exposure in the TSST-G.

### Psychological stress responses

**Figure 4:**
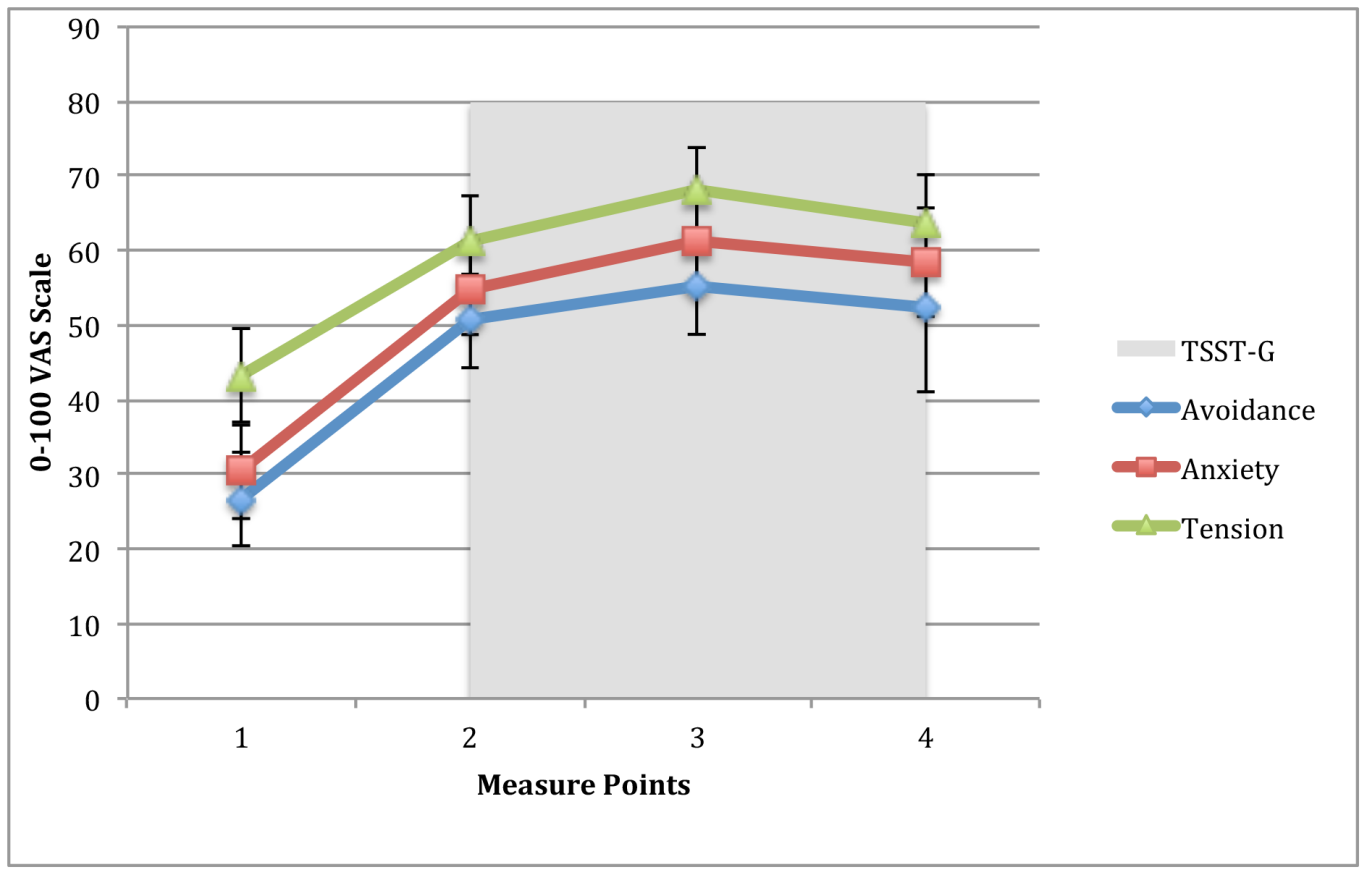
Visual analogue scales (VAS) completed before and during the TSST-G. The scales ranged from 0–100, and participants were told that 0 was no feeling of discomfort and 100 represented the worst feeling of avoidance: “To what extent do you want to leave this setting?” Anxiety: “To what extent do you feel uncomfortable in this setting?” And tension: “To what extent do you feel tension in your body right now?” Error bars represent 95% confidence intervals.

In accordance with the autonomic responses, but in contrast to the saliva cortisol responses, our participants indicated a stressful appraisal of the TSST-G exposure phase. Our participants rated their experience during the experiment on visual analog scales, using a scale of 0–100. The averaged rating of avoidance increased from 26.7 points (95%CI, 22.3–33.1) pre-exposure, to 55.4 points (95%CI, 48.9–63) during exposure. The rating of anxiety increased from 30.4 points (95%CI, 24.1–36.8) to 61.2 points (95%CI, 54.7–67.8) and tension increased from 43.3 points (95%CI, 37–49.6) to 68.1 points (95%CI, 55.6–67.3) (Figure 4).

## Discussion

This is the first description of a possibly blunted cortisol response in a heterogeneous group of patients on long-term sick leave. Our participants were exposed to a well-established, standardized psychosocial stressor, but failed to mount an adequate neuroendocrinological response (maximal difference of 26,7%). In contrast to this, their assessment of the Trier Social Stress Test for Groups (TSST-G) on VAS scales indicated that they in fact were stressed by the experiment, and heart-rate monitors showed a significant sympathetic activation collaborating this. Further, the participants did not show any marked neuroendocrinological recovery when measured for 60 minutes after ending the TSST-G.

The lack of variability in the cortisol response was not explained by reported use of medication, self-reported sleep problems, chronic fatigue, chronic pain, anxiety, depression, nor the presence of one or several SCID-I diagnoses. The only variable that had any effect on variation was being a current smoker, which is in line with studies showing habitual smokers have a decreased cortisol responsiveness to psychosocial stress [39]. However, the smokers only demonstrated an *even more* blunted response. The increase of free saliva cortisol expected from the TSST-G was not present in any of our participants.

Four main arguments increase the validity of our current results. First, the Trier test is well documented and causes a 200–500% cortisol increase in 75–85% of all participants [13], [24]. Second, rigidly designed studies have shown the measurement of adrenocortical activity through cortisol to be highly predictive of psychosocial stress [41]. Third, a recent review concluded that saliva cortisol is a reliable and valid measure of the biologically active component of cortisol [39]. Fourth, a lasting dysregulation of the HPA-axis would have several negative health consequences, perhaps the most prominent being a feeling of fatigue [2].

Our population consists of heterogeneous participants on long-term sick leave, referred to vocational rehabilitation, which is interesting when we interpret the current results. In Norway we have seen a 69% rise in people on long-term sick leave due to exhaustion/fatigue in the last 12 years [42]. Several studies have tied a dysregulation of the HPA-axis and a blunted response to standardized stressors to self-reported feelings of fatigue and exhaustion [8]. As the main function of cortisol is to prepare peripheral organs for action through increasing metabolism and releasing energy, the observed lack of cortisol responses may be a key factor to explain the high prevalence of fatigue in the population on long-term sick leave.

Seventy-nine percent of our participants reported clinically significant mental and physical fatigue lasting for more than 6 months. Seeing HPA-axis dysregulation as a common factor in patients on sick leave, manifesting through decreased energy and fatigue is in line with studies looking at these symptom-categories independently. However, observing the same effect, or lack there-of, in a group with different diagnoses and medical history is a cause for discussion. Kristenson et al. [12] outlined prolonged activation as causing lack of cortisol response and reactivity in their theoretical review of socioeconomic health differences. They theorized that successful coping leads to adequate responses, followed by relaxation and reduced activation. Lack of coping, helplessness, and/or hopelessness over time will lead to sustained activation and inability to recover. This state of prolonged activation would have several negative health effects, such as insulin resistance, persistent inflammation, infections and poor lifestyle choices [12].

When interpreting these results, it could indeed be argued that the TSST-G could have failed to cause stress in our participants. However, when comparing results with a TSST-G study on healthy participants using identical VAS-scales, our participants reported twice as much increase on anxiety, tension and avoidance from pre-exposure to exposure [24]. HPA-axis activity has been linked specifically to such ego-threatening psychosocial stress [43], and VAS scales are considered the gold standard when measuring psychosocial stress during exposure [37]. The procedure for TSST-G is adamant on the participant choosing a job they really want for the interview, as to ensure task engagement. It is likely that the engagement is even stronger in our population because they are selected with a personal goal of returning to work. The results from VAS-scales corroborate this.

Popular stress theories emphasize the concept of coping expectancy, and how this is crucial when regulating physiology in an effective and adequate manner [11]. It is tempting to speculate whether this could explain the observed results. The job-application part of the TSST-G would entail some of them preparing for an interview for a similar job as the one they would actually apply to in real life. Recent years have seen a development in how we understand transitions between work and benefits. Investigating a similar population to ours, Øyeflaten et al. [44] showed that during a 4-year follow-up, the average number of transitions was 3.7 (range 0–18) in a population on long-term sick leave. Falling in-and-out of work four times in four years would involve several negative coping experiences and likely affect patients' outcome expectancy.

### Limitations

Our results show an interesting psychoneuroendocrinological feature in participants on long-term sick leave. However, this study also has limitations. The foremost being the lack of matched healthy controls. Without a control group being subjected to exactly the same experimental procedure, in exactly the same location, we cannot make clear cut inferences about differences to a normal population. However, this study does not compare actual nmol/l values with previous studies, but rather high-low differences by percentages as measure of variability. The ability to respond and recover measured through cortisol is *the* factor of interest in stress theories, making a comparison by percentage of variation highly relevant.

It could also be considered a limitation that this study uses the short, group version of the TSST, rather than the “full” TSST. However, the protocol and administration of the Trier test in a group setting is thoroughly validated through several studies [24], [25]. A third limitation is the high percentage of women in this study. There are clear differences in how cortisol is regulated in men and women, and adult women usually show lower cortisol and sympathetic responses than men of the same age [43]. We found no statistical differences though, when participants were tested for sex and saliva cortisol variability, indicating no specific sex bias.

Moreover, we do not have complete control of all the social factors influencing the experimental session. For instance, a group dynamic would have formed in the preparation phase, possibly influencing the outcome without our knowledge. Finally, the studies listed in the discussion as comparison [24] have discrepancies with regard to culture, gender distribution and socioeconomic differences.

## Concluding remarks

Despite these limitations, our participants' rather specific failure to respond adequately to a stress experiment suggests a lack of cortisol reactivity as a biomarker of the pathway between sustained activation and sick leave. Previous studies have speculated as to whether a population on sick leave would have such pronounced effects [10]. Genetic studies have also suggested a biological disadvantage in catabolic stress responses for patients struggling with anxiety and depression [18].

This is the first study to suggest a lack of cortisol reactivity in patients on long-term sick leave and by doing so also supports highly cited appraisal theories of stress. Future studies should utilize a similar approach to investigate treatment effects on patients on long-term sick leave, including a control group, and possibly use genetic markers as to assess biological vulnerability.

## Supporting Information

Appendix S1 **Extended protocol description TSST-G.**
(DOCX)
