# Supplementary material for: The Role of Stress in Absenteeism: Cortisol Responsiveness among Patients on Long-Term Sick Leave
Source: PLoS One. 2014 May 2;9(5):e96048. doi: 10.1371/journal.pone.0096048 (PMC4008526; doi:10.1371/journal.pone.0096048)
Supplement: Appendix S1 — Extended protocol description TSST-G. (DOCX) [file pone.0096048.s001.docx]

## Appendix

#### Extended protocol description TSST-G

Job interview: This interview would include a 2-minute speech focusing solely on their personal qualities. The choice of job was tailored to the participants’ career wishes to ensure engagement. Further, they were told that presentation would be held in front of an expert panel consisting of specialists in non-verbal behavior. The participants were also told that a video camera and a microphone would record their performance in order to analyze body language and verbal behavior after the experiment was completed. One minute before the preparation phase was over the first saliva sample was collected along with three VAS scales and the heart rate monitor was started. Using the numbers, each presentation was randomly chosen by the test panel. If the participant finished his/hers presentation before the two minutes were up, the spokesman of the test panel would first wait several seconds, before stating “*You still have some time left*”. If this elicited no response, follow-up questions could be “*Which personal qualities would your friends and family use to describe you as a person?*” or “*Why do you think you are suitable for this job?*” After all of the participants finished their presentation, a second saliva sample was taken along with three VAS scales.

Calculation task: The calculation task involved subtracting the number 16 from a number given by the test panel (3330, 3314, 3298). They were told to calculate as fast and correctly as possible, while voicing their calculations out loud. If they miscalculated, the spokesperson would notify them of this by stating: “*Wrong! Begin from 3xxx*”. If the calculation was correct, the test panel gave no feedback and the participant would continue subtracting. Each participant spent 1 minute and 20 seconds on the arithmetic task. The third saliva sample was taken at the after the arithmetic task along with three VAS scales. This signified the end of the *exposure phase* and the heart rate monitor was stopped.
